# Flexible Resource Allocation-Efficient Water Use Strategies Facilitate Invasion of Invasive Vine *Sicyos angulatus* L.

**DOI:** 10.3390/biology13060392

**Published:** 2024-05-29

**Authors:** Qingmin Pan, Chenyang Xue, Lin Meng, Ying Gao, Mengyang Yu, Lin Geng, Ping Guan, Bo Qu

**Affiliations:** 1 College of Biological Technology, Shenyang Agricultural University, Shenyang 110016, China; 2 Key Laboratory of Pollution Ecology and Environmental Engineering, Institute of Applied Ecology, Chinese Academy of Sciences, Shenyang 110016, China; 3 Yixian Water Conservancy Affairs Service Center, Yixian 121100, China

**Keywords:** invasive plants, vine, photosynthesis, root pressure

## Abstract

**Simple Summary:**

The invasive vine *Sicyos angulatus* L. destroys the natural ecosystem of invaded areas and seriously interferes with normal agricultural production. Understanding the growth and development characteristics of *S. angulatus* is necessary in order to explore its invasion mechanisms and implement appropriate prevention and control measures. Flexible resource allocation and efficient water use strategies promote the growth and development of *S. angulatus*. In addition to conventional pesticides and aboveground removal measures, strengthening the removal of the root system of *S. angulatus* helps to improve control efficiency.

**Abstract:**

The invasive vine *Sicyos angulatus* L. destroys the natural ecosystem of invaded areas. Understanding the differences in growth and development between *S. angulatus* and other plants is necessary to explore the invasion mechanisms of *S. angulatus* and implement appropriate prevention and control measures. Thus, this study compared the growth, photosynthesis, and root characteristics of invasive liana *S. angulatus* and other three vine plants, *Ipomoea nil* (L.) Roth, *Ipomoea purpurea* (L.), and *Thladiantha dubia* Bunge, at different growth stages: seedling, flowering, and fruiting. The results showed that the total biomass of *S. angulatus* in the fruiting stage was 3–6 times that of the other three plants, and the root biomass ratio and root–shoot ratio decreased throughout the growth stage. Throughout the growth stage, the total leaf area of *S. angulatus* was significantly higher than that of the other three plant types, and the specific leaf area of *S. angulatus* at the seedling and flowering stages was 2.5–3 and 1.4–3 times that of the other three plants, respectively. The photosynthetic rate, stomatal conductance, and transpiration rate of *S. angulatus* at the fruiting stage were significantly higher than those of the other three plants, and its water use efficiency was higher than that of the other three plants at the three growth stages, indicating its strong photosynthetic capacity. The root activity and root pressure of *S. angulatus* were also significantly higher than those of the other three plants at the seedling and flowering stages. These results show that *S. angulatus* flexibly allocates resources to its aboveground parts during the growth stage to ensure that the plant obtains the space necessary for its growth and development and that with the help of higher root pressure and root activity, *S. angulatus* can maintain higher photosynthesis and water use efficiency with fewer resources. Therefore, the prevention and control of *S. angulatus* requires a combination of aboveground and underground measures. Spraying conventional weedicide/herbicide and manually removing aboveground plants may lead to its resurgence.

## 1. Introduction

Biological invasion has a serious impact on biodiversity, ecosystem function, human health, and sustainable economic development and has become the focus of environmental science and ecology research [1,2]. From 1970 to 2017, the direct and indirect annual average economic costs caused by biological invasions worldwide were as high as USD 26.8 billion and are projected reach USD 46.8 billion by 2017 [3,4]. China has a vast territory, complex and diverse ecosystems, rich biodiversity, a long history of agricultural activities, and rapid economic development. China is also more vulnerable to invasion by invasive alien species. From 1998 to 2018, the number of alien invasive plants in China increased from 58 to 515 [2]. Therefore, analyzing and comparing the differences in growth and development between invasive and native plants and exploring the invasion mechanisms of invasive plants will help formulate prevention and control measures with economic, ecological, and social benefits, reducing the harm caused by invasive plants.

Invasive alien plants can seriously harm agriculture in invaded areas. For example, the invasive alien plant *Alternanthera philoxeroides* (Mart.) Griseb. can spread rapidly in rice fields because of its clonal reproductive characteristics, and a single ramet will not weaken its growth and developmental ability [5]. *A. philoxeroides* covers the surface of rice fields and affects rice growth and development [6]. In every 1 m^2^ of farmland, when the number of alien invasive *Xanthium strumarium* L. reaches 1–2 plants, the yield of sunflower decreases by 30–40% and the yield of maize decreases by 80% [7]. When *X. strumarium* coexists with *Carthamus tinctorius* L., the biomass and seed yield of the latter decrease by more than 90% and 40%, respectively [8]. These exotic herbaceous plants in the aforementioned studies often have developed roots, large crowns, and rapid growth abilities and can compete with farmland crops for limited resources in a short period, realizing significant advantages [8]. Notably, most of the literature on the growth and development characteristics and potential hazards of invasive plants has focused on herbaceous plants that are not invasive vines.

Vines, also known as climbing plants, are rooted in the soil and must rely on external support to grow vertically or horizontally to a certain height [9]. Similar to other types of invasive herbaceous plants, vines significantly affect the economy and environment [10]. Strong environmental adaptability and growth strategies promote the invasion and growth of invasive vines [11]. Vines use fewer resources (biomass) for root support functions and allocate more resources (biomass) for stem elongation and leaf area expansion than herbaceous plants do, and these adaptations ensure that vines can explore larger growth spaces and obtain large amounts of light resources, promoting their invasion [12]. For example, the invasive plant *Ipomoea cairica* (L.) Sweet hinders the growth and photosynthesis of host plants by covering and winding, forming a single dominant population in the invaded area, seriously affecting the biodiversity and ecological landscape of the spreading area, and directly reducing the yield and fruit quality of the orchard [13]. *Dioscorea bulbifera* L. is an invasive species in Florida (United States) and grows rapidly. It can not only block the infiltration of sunlight with the help of a large canopy, but also divide along many branches to increase the pressure on other plants, leading to their death [14]

Several studies have investigated the climbing methods, photosynthetic characteristics, and biomass allocation strategies of vines in response to environmental changes [12,15,16]. However, few studies have investigated the role of roots in the resource allocation, growth, and development of vines. Studies have shown that vines have high transpiration rates, high pre-dawn water potential, and low osmotic adjustment ability; therefore, the root depth of vines in tropical forests is deeper than that of trees [17,18]. Because vines require few structural support functions, their root biomass accounts for a relatively low proportion, and large-area leaves and long-distance trunks require vines to have perfect nutrient and water supply. Roots play an important role in the growth and development of vines; therefore, studying the root traits of invasive vines will help elucidate their invasiveness and invasion mechanisms.

*Sicyos angulatus* L. (*Sa*), a Cucurbitaceae annual herbaceous climbing herb native to North America, has the biological characteristics of climbing, strangling, reproduction, and adaptation [19]. Notably, the growth of merely one thorn melon can harm the growth of corn in the range of 333 m^2^ [20]. In addition to crops, *Sa* can seriously affect the growth and development of woody and herbaceous plants. Studies have confirmed that in an area of 4 hm^2^, *Sa* can completely cover hundreds of trees and shrubs, and ground herbs have difficulty growing [21]. *Sa* and three other vines, *Ipomoea nil* (L.) Roth (*In*), *Ipomoea purpurea* (L.) Roth (*Ip*), and *Thladiantha dubia* Bunge (*Td*), were used to conduct this study. We aimed to measure and compare the growth, photosynthesis, root activity, and root pressure characteristics of the four plants at three growth stages: vegetative, flowering, and fruiting. We hypothesized that (1) the invasive vines *Sa* would have different growth and biomass allocation strategies than other three vine plants; (2) that these strategies would ensure that *Sa* has high photosynthesis and effective water use capacity, and (3) that the root system of *Sa* plays an important role in maintaining plant growth and water use. This study contributes to the literature by providing a theoretical basis for revealing the growth and developmental characteristics of *Sa*, elucidating its invasion mechanisms and exploring effective prevention and control measures for *Sa*.

## 2. Materials and Methods

### 2.1. Study Area

The experimental site was located in a greenhouse (8 m × 30 m) at the Teaching and Research Base of Shenyang Agricultural University. *Sa* fruits were collected from a cornfield near Shugang Road (39°23′59″ N, 122°23′59″ E), Pulandian District, Dalian City, Liaoning Province, on 15 October 2021. The *Td* fruit was collected in Shenyang (41°56′25″ N, 123°38′50″ E), Liaoning Province, on 28 October 2021. *In* and *Ip* seeds were collected from Zhuanghe (Dalian City, 39°39′43″ N, 122°55′1″ E), Liaoning Province, on 29 October 2021.

### 2.2. Seeds

**Figure 1:**
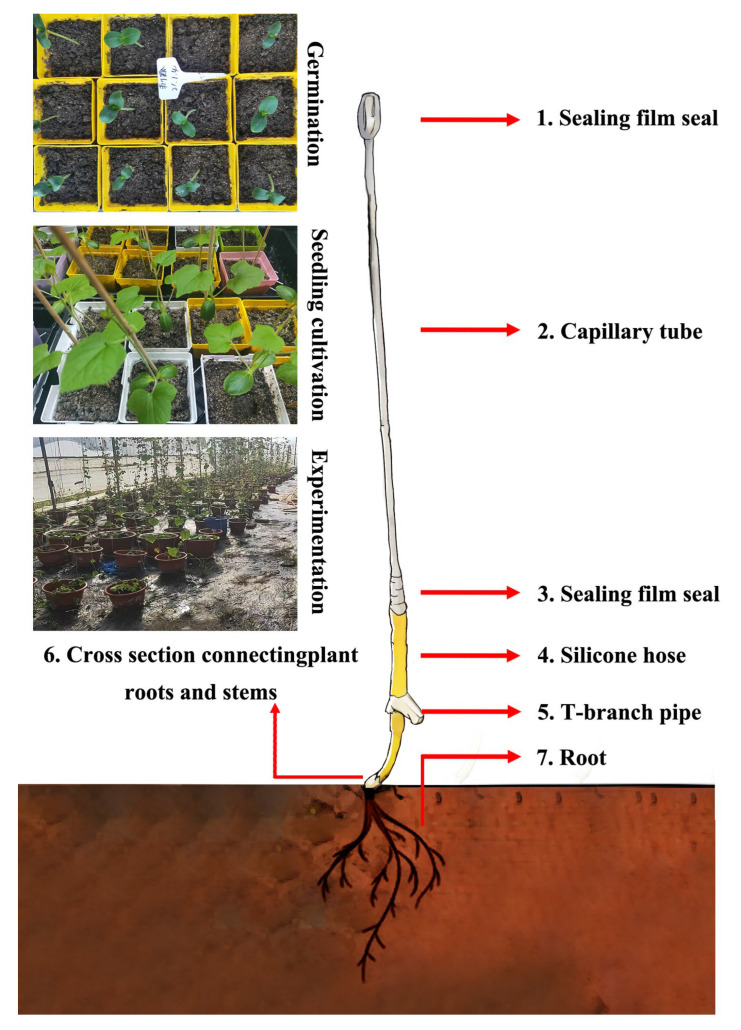
Diagram of root pressure measuring device.

The collected seeds were dried indoors, stored in a refrigerator at 4 °C, and germinated using the wet-glass method. Next, the seeds were disinfected in a 10% sodium hypochlorite solution for 10 min, washed five times using distilled water, immersed in warm water (55 °C) for 4 h, wrapped with gauze, placed in a self-sealing bag, and placed into a light incubator at 25 °C (LB-300-GB, Shanghai Yuejin Medical Device Factory, Figure 1). After 3–4 d, the germinated seeds were transferred into a plug for raising seedlings (Figure 1). After 7 d of growth in the seedling tray, 120 plants were selected for each plant type and transplanted into a greenhouse flowerpot (30 cm × 20 cm; for climbing vines, a rope was tied to the top of the greenhouse, and the other end was buried in the flowerpot), with one plant per pot (Figure 1, Supplementary Figure S1). The greenhouse temperature was room temperature, the relative humidity was maintained at 65%, and the pot soil moisture was maintained at 80 ± 4.6% (measured daily by a portable soil detector). At the teaching and research base of Shenyang Agricultural University, the soil was brown loam. In this study, a soilless seedling nutrition matrix (Shenyang Fanyu Horticultural Technology Co., Ltd., Jiaxing, China) and vermiculite were evenly mixed at a ratio of 3:1:1 and used to ensure that the soil moisture content was 60%. The growth and developmental characteristics of the four plants were measured at the vegetative, flowering, and fruit stages. Ten pots were established per group.

### 2.3. Measurements of Leaf Area, Photosynthetic Characteristics, and Water-Use Efficiency

The leaf areas of all the leaves at the top, lower top, middle, lower middle, and bottom of the four plants were measured using a portable laser leaf area meter (CI-203, China) during the vegetative, flowering, and fruiting stages. The net photosynthetic rate (*P_n_*), stomatal conductance (*G_s_*), and transpiration rate (*T_r_*) of the plant leaves were measured using a Li-6400 portable photosynthesis system (Li-COR, Lincoln, NE, USA). The light intensity of the leaf chamber was set at 1500 µmol·m^−2^·s^−1^ (saturated light intensity was determined by measurement), the CO_2_ concentration in the reference chamber was 380 ppm, the leaf temperature was the chamber temperature, and the relative humidity was 65%. Before measurement, the plants were fully induced under saturated light for 30 min, and the data were read after they were stable. Recently matured healthy leaves can reflect the maximum photosynthetic capacity of the plant and were thus selected for this study [22]. The formula for water use efficiency (WUET) was WUET = *P_n_*/*T_r_*, where *P_n_* is the net photosynthetic rate per unit leaf area, and the unit is μmol·m^−2^·g^−1^, while *T_r_* is the transpiration rate per unit leaf area, and the unit is g·m^−2^·h^−1^.

### 2.4. Determination of Root Pressure and Root Activity

The reference bubble manometer method (bubble manometer) [23] was conducted based on the original method (Figure 1): The root pressure measuring device was installed after the plant stem was cut directly from the ground between 2 and 5 cm, and after 1 h, the root pressure was collected by measuring the length of the capillary hose. The specific operation method used was as follows: A silica gel hose consistent with the thickness of the plant rhizome was used. One end was connected to the tee, and the other end of the tee was connected to the capillary hose. After pouring distilled water into one-third of the capillary hose, the capillary hose was sealed. Before sunrise, approximately between 03:00 and 04:00, on the day of the experiment, the selected plants were cut and leveled at 2–5 cm from the rhizome with fruit branches, and a silicone hose was sleeved on the stem. The silicone hose was connected to the stem, the middle of which was sealed with a sealing film, and the length of the capillary hose bubble was measured after allowing it to stand for 1 h. The formula for calculating the root pressure was *P_x_* = 100 × [(*L_atm_*/*L_pd_*) − 1], where *P_x_* is the root pressure or sample xylem pressure (kPa), *L_atm_* is the bubble length in the capillary hose balanced with the external atmospheric pressure, and *L_pd_* is the length of the bubbles in the capillary tube before reaching equilibrium with the external atmospheric pressure. The root activity was determined using the TTC method [24].

### 2.5. Statistical and Data Analyses

SPSS 22.0 software (IBM, New York, NY, USA) was used to compare the differences in plant growth and development (total biomass, biomass ratio, root–shoot ratio, leaf area, and specific leaf area) characteristics, photosynthetic characteristics, and root activity and root pressure for the different plants and different growth stages (one-way analysis of variances [ANOVA] with LSD tests). All images were rendered using GraphPad Prism 8 (GraphPad Software, La Jolla, CA, USA). The aforementioned analysis was tested for significance at *p* < 0.05; *p* ≥ 0.05 represented no significance.

## 3. Results

### 3.1. Compared with the Other Three Plants, Sa Can Maintain Rapid Growth and Development

The total biomass of the four vines continued to increase as the growth stage progressed (Table 1). At the seedling, flowering, and fruiting stages, the plants with the highest total biomass were *Td*, *Ip*, and *Sa*, respectively, which were significantly higher than those of the other plants (Table 1, *p* < 0.05). However, the growth rate of the invasive plant *Sa* reached 293% during the flowering period, significantly higher than that of *In* and *Td*, and the growth rate during the fruit period reached 1051%, significantly higher than that of the other plants (Table 1). The results of the biomass proportion of each part of the plant showed that the proportion of root and leaf biomass of the four vines decreased gradually with the growth stage, and the stem showed the opposite growth trend (Table 1). At the fruit stage, the root biomass ratio of *Sa* was 2.89%, significantly lower than that of the other plants (Table 1, *p* < 0.05). The stem and leaf biomass ratios were 67.32% and 29.77%, respectively, significantly higher than those of the other plants (Table 1, *p* < 0.05). During the three growth stages, *Sa* showed continuous growth ability, a higher proportion of stem and leaf biomass, and many stem support structures; leaves helped increase the coverage area of *Sa* above ground and obtain sufficient light and living space. Notably, plants with a larger biomass and a higher proportion of aboveground parts often require strong roots to fulfill the plant’s water and nutrient requirements, whereas the *Sa* root biomass showed a diametrically opposite trend (Table 1, Supplementary Figures S2–S4), suggesting that *Sa* has a unique growth strategy.

**Figure 2:**
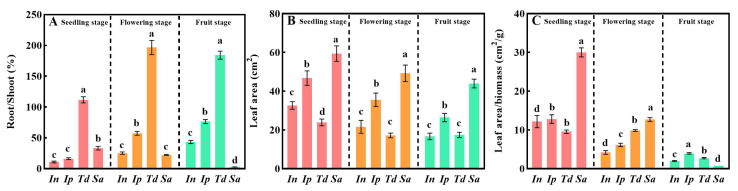
Characteristics of root–shoot ratio, leaf area, and specific leaf area of four vines at different growth stages. (**A**): characteristics of root–shoot ratio of four vines at different growth stages; (**B**): characteristics of leaf area of four vines at different growth stages; (**C**): characteristics of specific leaf area of four vines at different growth stages. Root/Shoot: root biomass/shoot biomass, shoot represents the aboveground biomass of the plant; *In*: *Ipomoea nil* (L.) Roth; *Ip*: *Ipomoea purpurea* (L.); *Td*: *Thladiantha dubia* Bunge; *Sa*: *Sicyos angulatus* L. Different lowercase letters indicate the significant differences among *In*, *Ip*, *Td*, and *Sa* under the same stage (*p* value < 0.05).

Consistent with the aforementioned conclusions, the root–shoot ratio of *Sa* was significantly lower than that of the other three plants during the three growth periods and showed a significant downward trend (Figure 2A, *p* < 0.05), which was opposite to the gradually increasing trend of the other three plants. In addition, the total leaf area of the four plants showed a downward trend with the change in the growth stage. However, the leaf area of the *Sa* plants was significantly higher than that of the other three plants (Figure 2B, *p* < 0.05). The leaf area reduction rate in the flowering and fruit stages was 17.05% and 10.76%, respectively, much lower than that of the other three plants (Figure 2B); the results for specific leaf area showed that the specific leaf area of the four plants gradually decreased with the growth stage (Figure 2C). The specific leaf area of *Sa* was significantly higher than that of the other three plants during the seedling and flowering stages (Figure 2C, *p* < 0.05). In general, a high specific leaf area indicates that plants have a larger leaf area per unit of material energy, which helps increase the area for photosynthesis and promote plant growth and development.

### 3.2. Photosynthetic Capacity of Sa Fruit Period Was Stronger Than That of In, Ip, and Td

**Figure 3:**
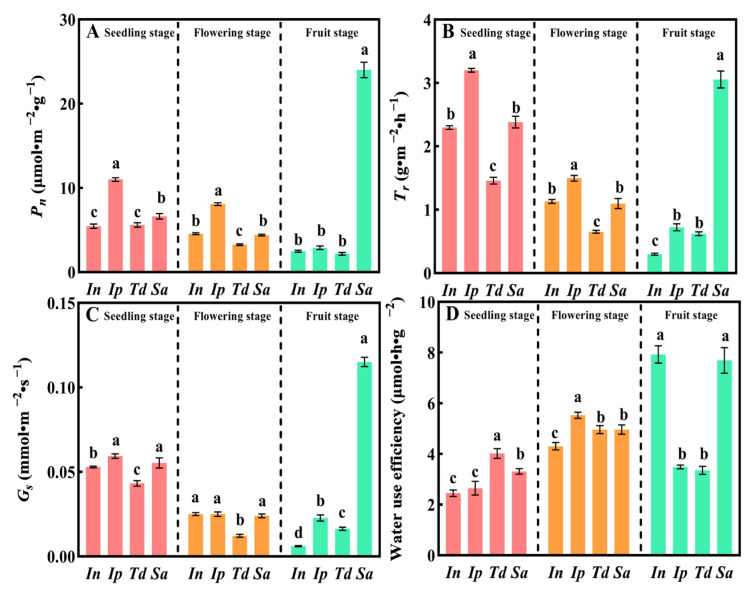
Photosynthetic characteristics of four vines at different growth stages. (**A**): the net photosynthetic rate of four vines at different growth stages; (**B**): the transpiration rate of four vines at different growth stages; (**C**): the stomatal conductance of four vines at different growth stages; (**D**): the water use efficiency of four vines at different growth stages. *In*: *Ipomoea nil* (L.) Roth; *Ip*: *Ipomoea purpurea* (L.); *Td*: *Thladiantha dubia* Bunge; *Sa*: *Sicyos angulatus* L. Different lowercase letters indicate the significant differences among *In*, *Ip*, *Td*, and *Sa* under the same stage (*p* value < 0.05).

The photosynthetic characteristics of the leaves of the four plants showed that the maximum net photosynthetic rate of *Sa* in the fruiting period was significantly higher than that of the other three plants (Figure 3A, *p* < 0.05), and the maximum net photosynthetic rate of *Sa* at the seedling and flowering stages was weaker than that of *Ip* (Figure 3A), which was consistent with the total biomass results of the four plants (Figure 3A). At the fruit stage, the *Tr* and *Gs* of *Sa* were significantly higher than those of the other three plants (Figure 3B–D, *p* < 0.05). Notably, the photosynthetic characteristics of *In*, *Ip*, and *Td* decreased gradually with the growth and development stages (Figure 3A–C), and *Sa* maintained a high photosynthetic capacity in the fruiting stage (Figure 3A–C). Thus, different from the other three plants, the rapid growth stage of *Sa* was located between the flowering and fruiting stages, and of the four plants, *Sa* had the longest photosynthetic cycle. Notably, the rapid growth and high transpiration rates of plants have high water use efficiency requirements. The water use efficiency of *Sa* was high across the three growth stages and gradually increased with the growth stage, playing an important role in its growth and development (Figure 3D).

### 3.3. Sa Had Higher Root Pressure and Root Activity Than In, Ip, and Td

**Figure 4:**
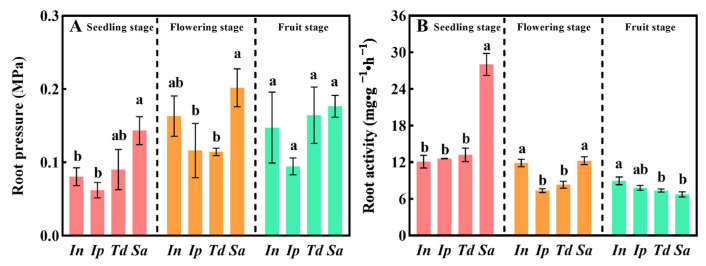
Root characteristics of four vines at different growth stages. (**A**): the root pressure of four vines at different growth stages; (**B**): the root activity of four vines at different growth stages. *In*: *Ipomoea nil* (L.) Roth; *Ip*: *Ipomoea purpurea* (L.); *Td*: *Thladiantha dubia* Bunge; *Sa*: *Sicyos angulatus* L. Different lowercase letters indicate the significant differences among *In*, *Ip*, *Td*, and *Sa* under the same stage (*p* value < 0.05).

The root pressure of the four plants gradually increased with the growth stage. At the seedling and flowering stages, the root pressure of *Sa* was significantly higher than that of the other three plants (Figure 4A, *p* < 0.05). At the fruiting stage, the root pressure of *Sa* was higher than that of the other three plants (Figure 4A). The root activity of *Sa* was significantly higher than that of the other three plants at the seedling stage (Figure 4B, *p* < 0.05). High root pressure and vitality help ensure the absorption and transport of water and nutrients during plant growth and development, which is crucial for vines.

## 4. Discussion

**Figure 5:**
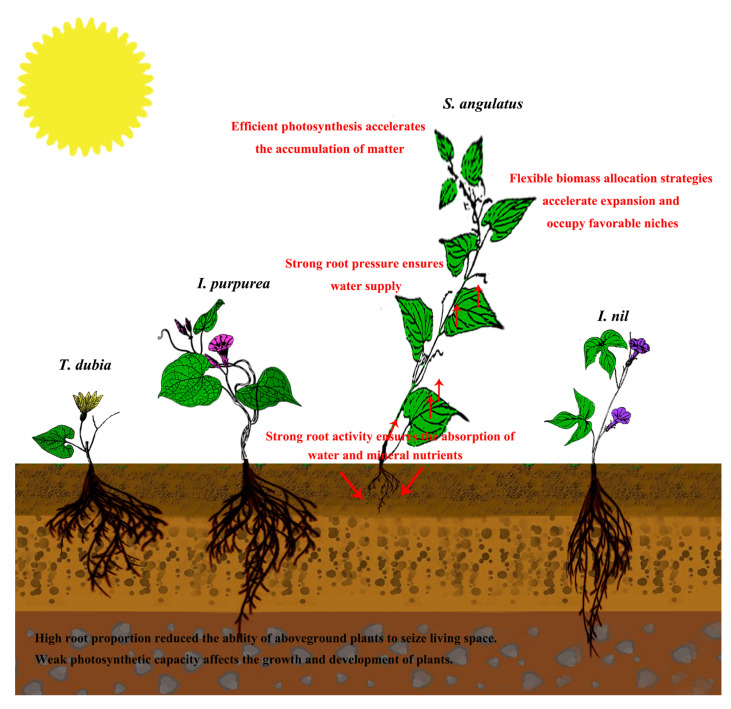
Growth strategy and invasion characteristics of *S. angulatus*.

The growth and development characteristics of herbaceous vines and other herbaceous plants significantly differ. As described in the preface, vines rely on external support to grow vertically or horizontally to a certain height, and the latter requires a certain degree of lignification [25]. Therefore, a primary means for invasive vines to harm native plants is climbing and covering, promoting invasion by destroying the photosynthetic and nutrient absorption capacities of the latter [26]. Therefore, invasive vines tend to have specific biomass allocation characteristics that may help them occupy dominant niches in different habitats (Figure 5). In this study, the invasive plant *Sa* showed a rapid growth rate and a great biomass advantage during the fruiting period (Table 1). Generally, rapid growth rates and biomass are the basic advantages of invasive plants, which help them quickly occupy suitable niches, seize favorable environmental resources, and accumulate the living space of native plants, promoting the formers’ growth and population expansion [27]. For example, invasive plants such as *Eupatorium adenophorum* Spreng. [28], *X. strumarium* [29], and *Ambrosia trifida* L. [30] have larger biomass and faster relative growth rates than native plants in the areas that they invade.

In this study, the results of biomass allocation investigations showed that the stem biomass ratio of the four plants increased significantly with the growth stage (Table 1). Some researchers have suggested that vines require few supporting structures and that their lower stem biomass ratio can ensure that they use more resources for leaves and reproduction [31]. However, a higher stem proportion contributes to rapid increases in vine length, which contributes to the invasion of plants to some extent [14]. The root biomass ratio and root–shoot ratio findings from this study showed that the three non-invasive vines continuously increased their proportion of roots across the growth stages, while the root proportion of the invasive plant *Sa* decreased continuously (Table 1); additionally, the proportion of leaf biomass and leaf area of the other three plants decreased more than that of the *Sa* plants (Table 1 and Figure 2C). The allocation strategy for plant biomass is significantly affected by natural environmental factors [32]. For example, when plants experience water stress, they may increase the allocation of resources to roots and stems to manage water deficits and reduce leaf loss [33], and under competitive conditions, *Vitis vinifera* L. usually increases the allocation of resources to roots and stems to seize growth space [34]. In the absence of soil nutrients, *E. adenophorum* and *E. odoratum* show high plasticity in response to changes in nitrogen nutrition. With an increase in nitrogen, the root-shoot and root biomass ratios of the two plants have been shown to decrease, and the leaf biomass ratio to increase. Low nitrogen conditions increase the biomass allocation of roots to fulfill their nutrient needs, and under high nitrogen conditions, biomass use for leaves increases to improve resource capture [27]. Our study was conducted in a controlled environment without the influence of nutrients, water, or competition. We speculate that the invasive vine *Sa* preferentially allocates resources to stems and leaves, which may be beneficial for the rapid growth of plants and thus the occupation of sufficient growth space (Figure 5).

With economic development and the intensification of globalization, the phenomenon of invasive vines in habitats disturbed by the external environment is becoming increasingly prominent [35]. Notably, simple external morphology and resource allocation strategies are insufficient to explain invasion mechanisms [36]. Physiological traits closely related to growth and development, but not directly related to climbing strategies, are worthy of in-depth exploration. Photosynthetic data showed that the photosynthetic characteristics of the invasive vine *Sa* at the fruit stage were significantly higher than those of the other three plants (Figure 3). Although several studies have shown that the photosynthetic rate of some invasive plants is usually higher than that of native plants [27,29,37], a higher photosynthetic rate in a single environment may be sufficient to promote the success of invasion, because higher carbon gain may increase the adaptability to the environment. However, in this study, the maximum net photosynthetic rate of *Sa* at different growth stages showed similar changes in biomass (Table 1 and Figure 3A). In addition, studies have confirmed that a high specific leaf area can increase the light-harvesting ability of plant leaves and promote a high growth rate [38]. In this study, the specific leaf area of *Sa* at the seedling and fruit stages was significantly higher than that of the other three plants (Figure 2C). We speculate that at the seedling and flowering stages, the higher leaf area and specific leaf area of *Sa* than those of the other three plants compensate for the former’s defects in photosynthetic rate, whereas the high light absorption and photosynthetic rate of *Sa* in the fruiting stage indicate that photosynthesis can be maintained for a long time during its growth cycle and that the long-term photosynthetic rate is conducive to increasing the biomass accumulation of plants. Vines are generally considered to have a higher average gas exchange capacity, that is, stomatal conductance, than climbing herbs and woody plants [39]. Owing to the large hydraulic limitation of vines, their stems must support a disproportionately large leaf area; therefore, their higher stomatal conductance helps them to maintain a normal water demand [40]. In this study, *Sa* showed a higher water use efficiency. At the fruiting stage, the water use efficiency of *Sa* was similar to that of *In*, and both were significantly better than that of *Ip* and *Td* (Figure 3D). Studies have suggested that having greater water use efficiency than other plants is an important factor for successful plant invasion [41].

In this study, the root biomass ratio and root–shoot ratio of *Sa* gradually decreased with the growth stage and were significantly lower than those of the other three plants at the fruit stage (Table 1 and Figure 3A). The stem structure of vines is long and has a narrow cross-section; thus, the water safety factor of plants is low and vulnerable to damage [31]. To avoid the formation of embolisms in the upstream stem and the limitation of water flow, the roots of vines must maintain a continuous water supply, and root pressure can reduce the water limitation of the aboveground part of the plant. Root pressure has been shown to be an important physiological mechanism for the phytoremediation of embolic catheters during water deficits [42,43]. Evidence suggests that the roots of some tropical [44] and temperate vines [45] may produce positive stem water pressures during the rainy season or at night. In the three growth stages, the root pressure of the invasive plant *Sa* was higher than that of the other three plants (Figure 4A), and except for the fruit period, the root activity of *Sa* showed a similar trend (Figure 4B). In some temperate vines, even with high transpiration, water stored in the roots may be involved in maintaining an increase in xylem water potential [46]. However, analyzing the morphology of plant xylems and other tissues and the growth rate of roots in different sectors is necessary to improve the understanding of the absorption and storage of water in the roots of vines. Based on the existing growth and physiological data, we speculate that *Sa* has a high specific leaf area and low transpiration rate at the seedling and flowering stages, which helps reduce water loss. Additionally, the high root pressure and root activity of *Sa* ensure water supply to the aboveground tissues of plants. In the fruiting stage, on the basis of maintaining a certain rate of photosynthesis in plants, *Sa* adopts low specific leaf area and high root pressure strategies to alleviate the water loss caused by a high transpiration rate (Figure 5).

## 5. Conclusions

In this study, the growth, morphology, photosynthesis, and root characteristics of four types of plants in the whole growth cycle were observed and compared, and the invasion characteristics of *Sa* were expounded from the perspective of aboveground and underground. This method not only helped reveal the invasion mechanisms of *Sa*, but also provided a theoretical basis for prevention and control strategies of *Sa* in agricultural production activities. We suggest that in addition to conventional pesticides and aboveground removal measures, strengthening the removal of the roots of *Sa* will help improve the efficiency of prevention and treatment.

## Figures and Tables

**Table 1:** Biomass characteristics of four vines at different growth stages.

Plant	Index	Seedling Stage	Flowering Stage	Fruit Stage
*In*	Total biomass (g)	5.03 ± 1.94 b	22.85 ± 2.86 b	31.85 ± 6.32 c
	Root ratio (%)	23.35 ± 3.98 d	17.52 ± 2.01 c	29.16 ± 3.37 c
	Steam ratio (%)	2.55 ± 0.41 c	53.61 ± 2.26 b	46.94 ± 4.00 b
	Leaf ratio (%)	74.10 ± 1.40 a	28.87 ± 0.69 a	23.90 ± 0.62 b
*Ip*	Total biomass (g)	4.97 ± 0.64 b	51.54 ± 7.03 a	63.62 ± 12.24 b
	Root ratio (%)	27.74 ± 5.19 c	36.22 ± 2.75 b	42.61 ± 2.33 b
	Steam ratio (%)	3.21 ± 0.52 c	52.7 ± 2.30 b	46.95 ± 2.27 b
	Leaf ratio (%)	69.05 ± 1.79 b	11.08 ± 0.20 c	10.44 ± 0.14 c
*Td*	Total biomass (g)	6.90 ± 1.54 a	6.99 ± 0.90 d	59.98 ± 10.46 b
	Root ratio (%)	49.77 ± 3.10 a	66.06 ± 4.25 a	65.29 ± 1.30 a
	Steam ratio (%)	15.27 ± 1.13 a	10.57 ± 1.13 c	23.84 ± 1.94 c
	Leaf ratio (%)	34.96 ± 0.51 d	23.37 ± 1.10 b	10.87 ± 0.32 c
*Sa*	Total biomass (g)	4.27 ± 0.82 b	16.80 ± 1.92 c	193.41 ± 29.88 a
	Root ratio (%)	41.99 ± 2.39 b	18.23 ± 0.59 c	2.90 ± 0.30 d
	Steam ratio (%)	7.74 ± 0.83 b	58.96 ± 1.95 a	67.33 ± 1.02 a
	Leaf ratio (%)	50.27 ± 0.73 c	22.81 ± 0.71 b	29.77 ± 0.28 a

*In*: *Ipomoea nil* (L.) Roth; *Ip*: *Ipomoea purpurea* (L.); *Td*: *Thladiantha dubia* Bunge; *Sa*: *Sicyos angulatus* L. Different lowercase letters indicate the significant differences among *In*, *Ip*, *Td*, and *Sa* under the same stage (*p* value < 0.05).

## Data Availability

Data can be made available upon reasonable request.

## Supplementary Materials

The following supporting information can be downloaded at: https://www.mdpi.com/article/10.3390/biology13060392/s1, Figure S1: The photos at different stages; Figure S2: Seedling stages; Figure S3: Flowering stages. Figure S4: Fruiting stages.
