# Supplementary material for: Flexible Resource Allocation-Efficient Water Use Strategies Facilitate Invasion of Invasive Vine Sicyos angulatus L
Source: Biology (Basel). 2024 May 29;13(6):392. doi: 10.3390/biology13060392 (PMC11200540; doi:10.3390/biology13060392)
Supplement: Supplementary file 1 [file biology-13-00392-s001.zip › biology-3017765-supplementary.pdf]

Supplementary Figure

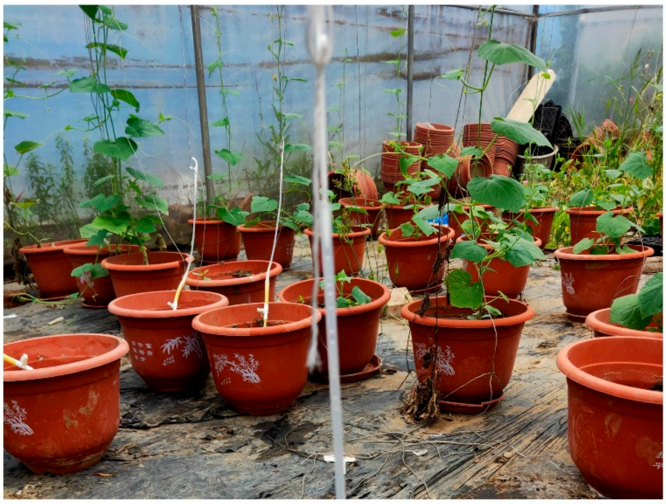

Seedling stage

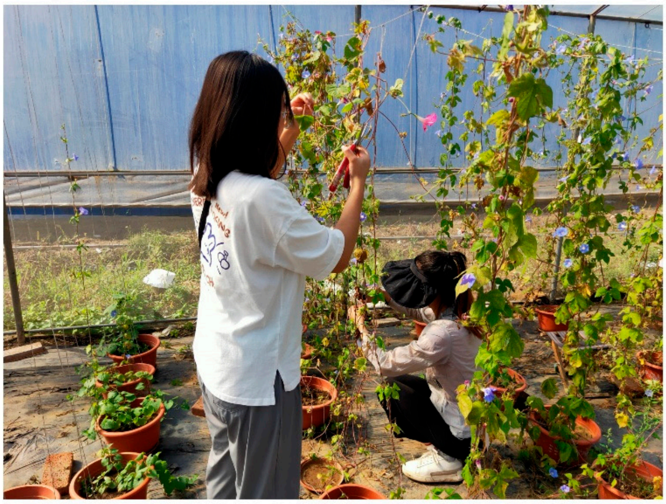

Flowering stage

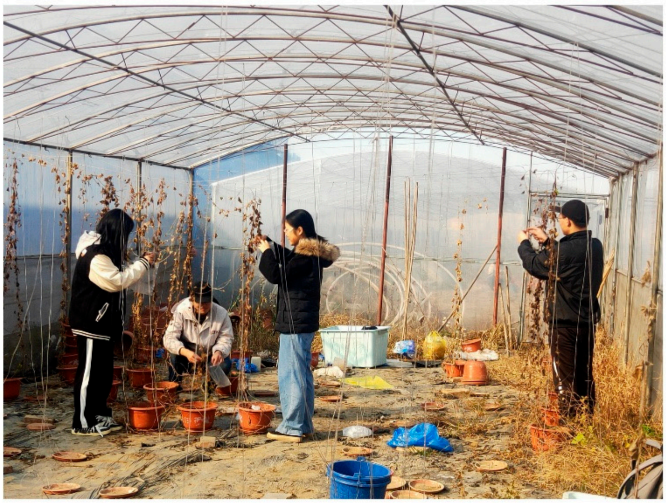

Fruit stage

(Photosynthesis at fruit stage was determined before leaf withered)

Figure S1. The photos at different stages.

Ipomoea nil* (L.) Roth (*In*)** **| *Ipomoea purpurea* (L.) Roth (*Ip*)** **| *Thladiantha dubia* Bunge (*Td*)** **| *Sicyos angulatus L. (Sa)

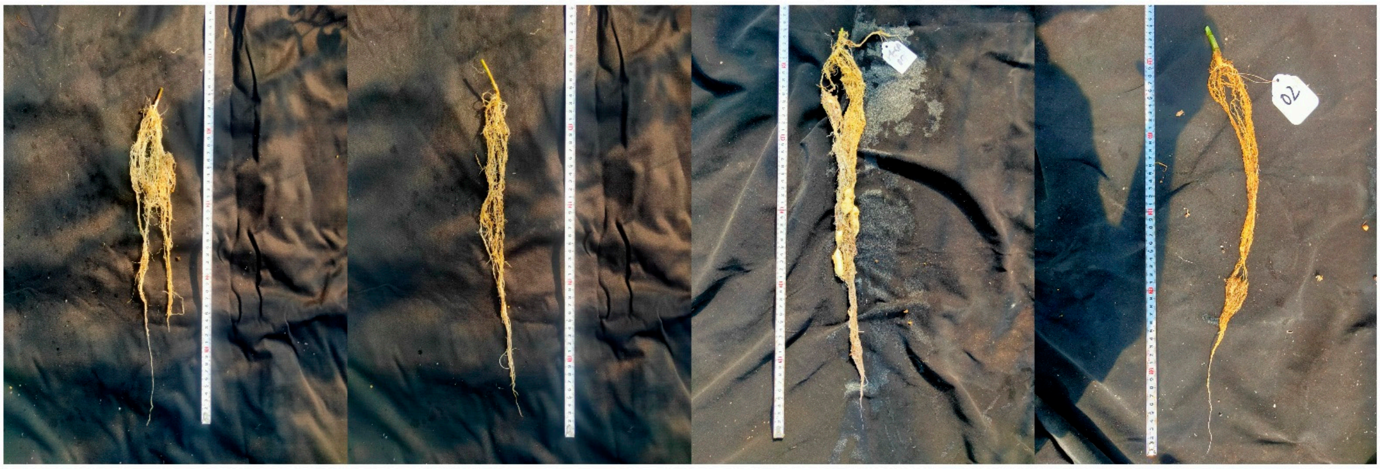

Figure S2 Seedling stages.

Ipomoea nil* (L.) Roth (*In*)** **| *Ipomoea purpurea* (L.) Roth (*Ip*)** **| *Thladiantha dubia* Bunge (*Td*)** **| *Sicyos angulatus L. (Sa)

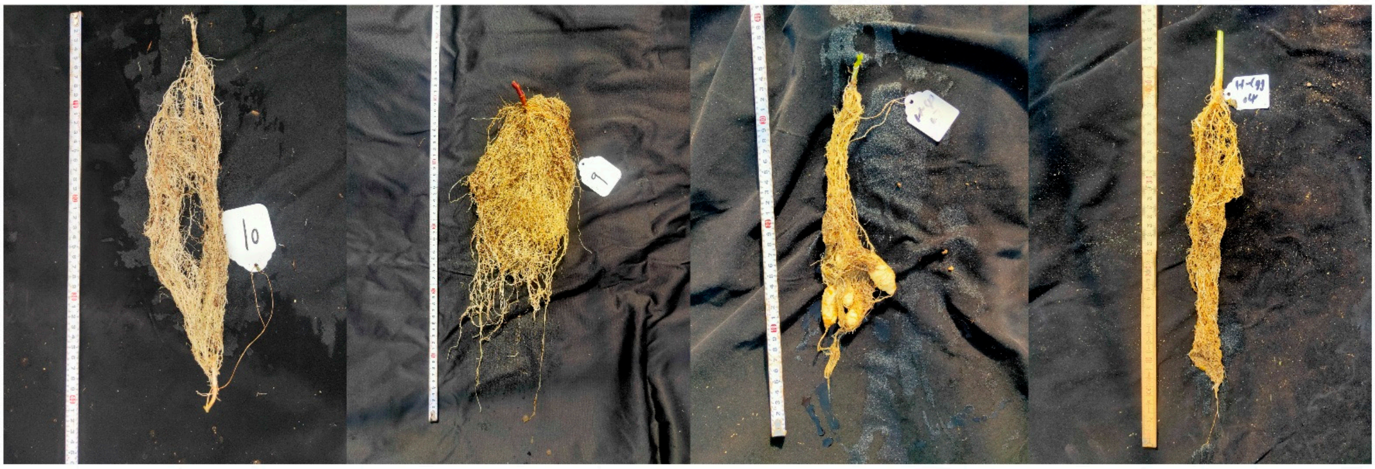

Figure S3 Flowering stages.

Ipomoea nil* (L.) Roth (*In*)** **| *Ipomoea purpurea* (L.) Roth (*Ip*)** **| *Thladiantha dubia* Bunge (*Td*)** **| *Sicyos angulatus L. (Sa)

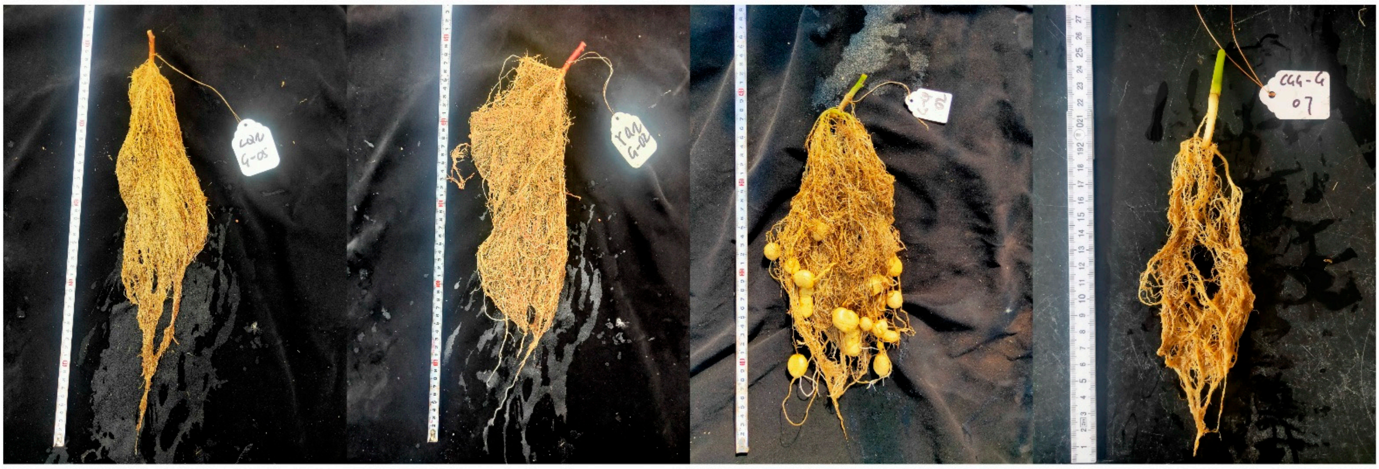

Figure S4 Fruiting stages.
